# Pediatric Intraosseous Cavernous Hemangioma of the Mandible: A Case Series Highlighting Diagnostic Challenges and Surgical Management

**DOI:** 10.7759/cureus.109626

**Published:** 2026-05-25

**Authors:** Subhas Chandra Debnath, Barnakshi Deka, Payal Poddar, Palak Kaul

**Affiliations:** 1 Oral and Maxillofacial Surgery, Regional Dental College, Guwahati, IND

**Keywords:** diagnostic challenge, hemangioma, pediatric pathology, surgical complication, vascular malformation

## Abstract

Intraosseous hemangioma of the mandible is a rare benign vascular tumor, accounting for less than 1% of primary bone tumors, and is exceptionally uncommon in pediatric and adolescent populations. Owing to its nonspecific clinical presentation and variable radiographic appearance, it is frequently misdiagnosed as an odontogenic lesion, posing a significant risk of intraoperative hemorrhage. We report three cases of intraosseous cavernous hemangioma of the mandible in an 11-year-old female, a nine-year-old male, and a 14-year-old female presenting with painless mandibular swelling and facial asymmetry. Radiographic features ranged from a classic sunburst pattern to multilocular radiolucency and osteolytic lesions with hypervascular soft-tissue components. Aspiration and computed tomography with angiographic evaluation played a crucial role in identifying the vascular nature of the lesions. All patients were successfully managed with segmental mandibulectomy following vascular control and appropriate reconstruction. Histopathological examination confirmed cavernous hemangioma in all cases. This case series highlights the importance of considering intraosseous hemangioma in the differential diagnosis of mandibular lesions in young patients and emphasizes the critical role of angiographic evaluation and preoperative planning in preventing life-threatening hemorrhagic complications.

## Introduction

Intraosseous hemangioma is a rare benign vascular lesion of the bone, accounting for a very small proportion of osseous tumors involving the maxillofacial region. Among jaw bones, the mandible is more frequently affected than the maxilla, with a predilection for the body and symphysis regions [1,2]. These lesions arise from proliferation of blood vessels within bone and are currently considered part of the spectrum of intraosseous vascular anomalies rather than true neoplasms [3].

Clinically, intraosseous hemangiomas often present as slow-growing, painless swellings and may remain asymptomatic for prolonged periods. However, in certain cases, they may exhibit features such as cortical expansion, tooth mobility, or bleeding, depending on their size and vascularity [2,4]. The absence of consistent clinical signs often leads to misdiagnosis, with these lesions frequently mimicking odontogenic cysts or tumors [4].

Radiographically, intraosseous hemangiomas demonstrate considerable variability, ranging from multilocular radiolucencies to mixed radiopaque patterns. Classical appearances such as “sunburst” or “honeycomb” patterns are not always present, further complicating diagnosis [4,5]. Because of their vascular nature, invasive procedures without prior diagnosis may result in severe hemorrhagic complications, highlighting the importance of careful evaluation [6].

Advanced imaging modalities, such as computed tomography and angiography, play an essential role in assessing lesion extent and vascular supply, thereby aiding treatment planning and enabling advanced techniques, such as virtual surgical planning and precise mandibular reconstruction [1,7]. The present case series aims to highlight the diverse clinical and radiographic presentations of intraosseous hemangioma of the mandible and to emphasize the importance of early diagnosis and appropriate management.

## Case presentation

Case 1

**Figure 1:**
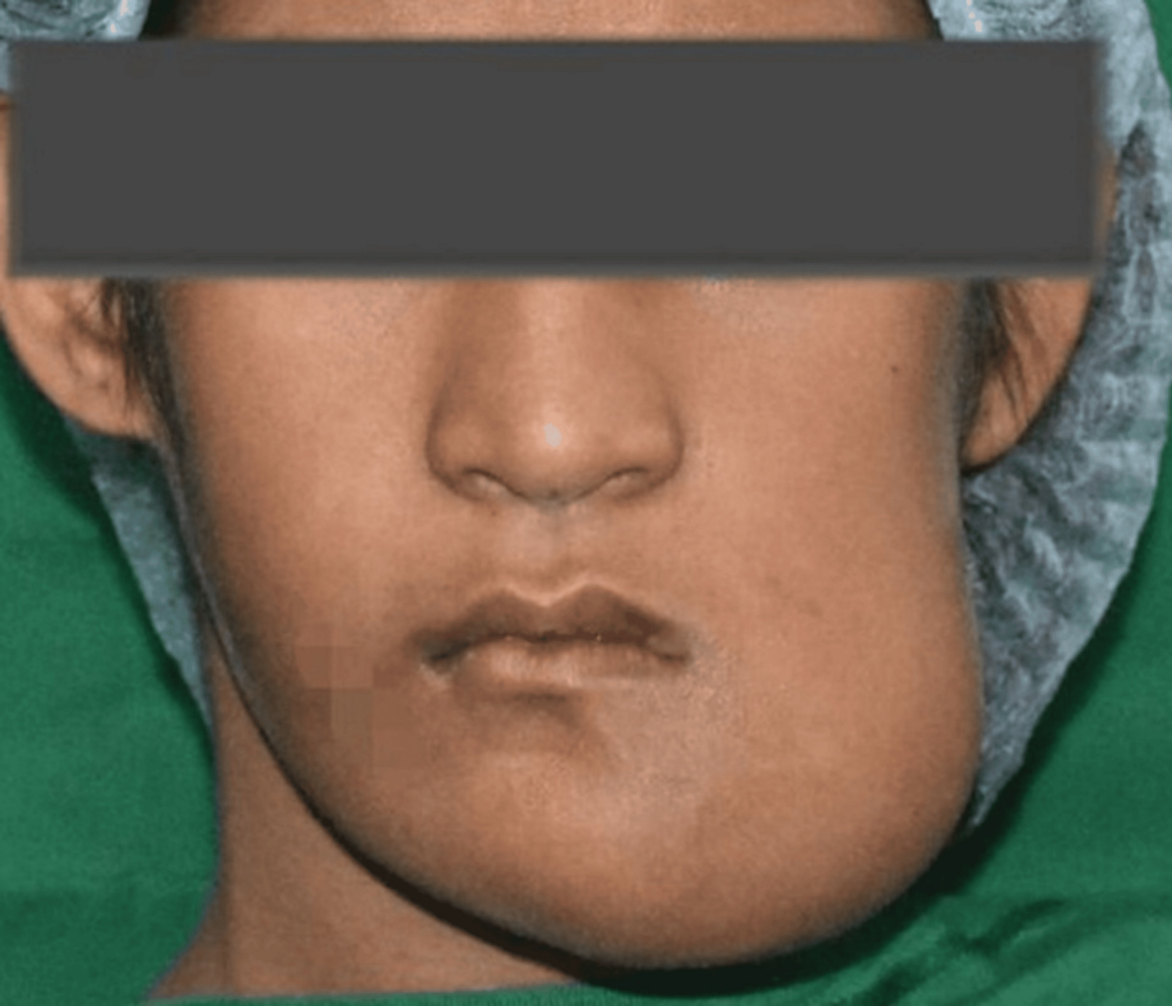
Preoperative extraoral clinical photograph showing facial asymmetry due to swelling involving the left lower third of the face.

An 11-year-old female presented with a gradually enlarging swelling involving the lower third of the face for approximately one year (Figure 1).

The swelling extended intraorally from the right canine to the left second molar region. Clinical examination revealed facial asymmetry due to a firm, non-tender, pulsatile swelling. Intraorally, buccal and lingual cortical expansion with displacement of teeth was noted; however, none of the teeth exhibited mobility. The overlying mucosa appeared intact, and no neurological deficit was detected.

**Figure 2:**
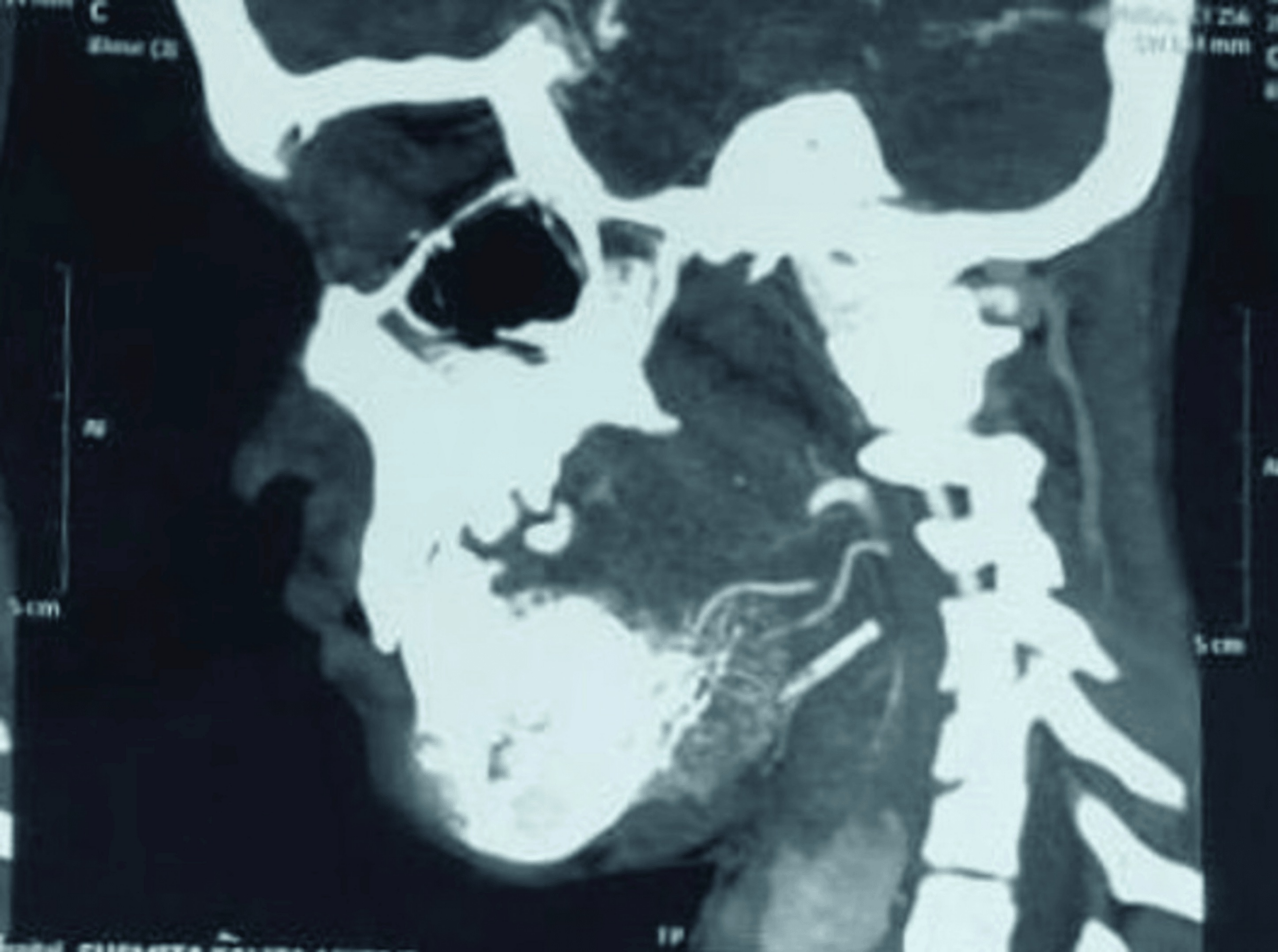
Sagittal CT angiogram showing the facial artery as a feeder vessel. Slice thickness: 1 mm at 1 mm interval.

Orthopantomogram revealed a radiating trabecular pattern resembling a “sunburst” appearance. Computed tomography demonstrated an expansile intrabony lesion with multiple vascular channels and cortical thinning measuring approximately 56 × 56 × 66 mm in greatest dimensions. Preoperative angiography confirmed multiple feeder vessels arising from the facial artery (Figure 2).

**Figure 3:**
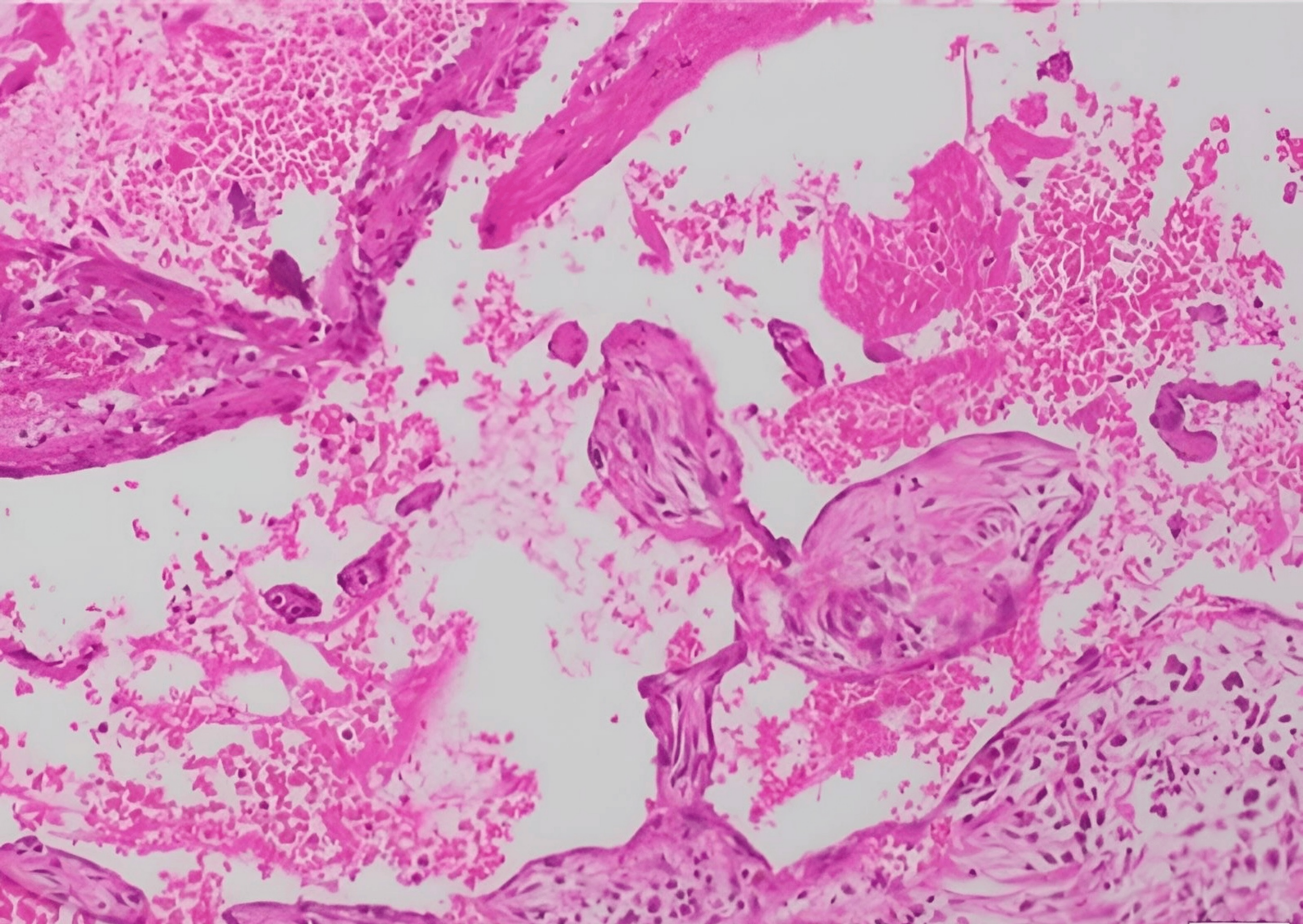
Post-operative HPE confirms cavernous hemangioma. Magnification: 10x. HPE: histopathological examination.

Surgical excision was planned following facial artery ligation to minimize intraoperative bleeding. Segmental mandibulectomy was performed under general anesthesia. Histopathological examination (HPE) revealed multiple cavernous vascular spaces lined by endothelial cells within trabecular bone, confirming the diagnosis of intraosseous cavernous hemangioma (Figure 3).

**Figure 4:**
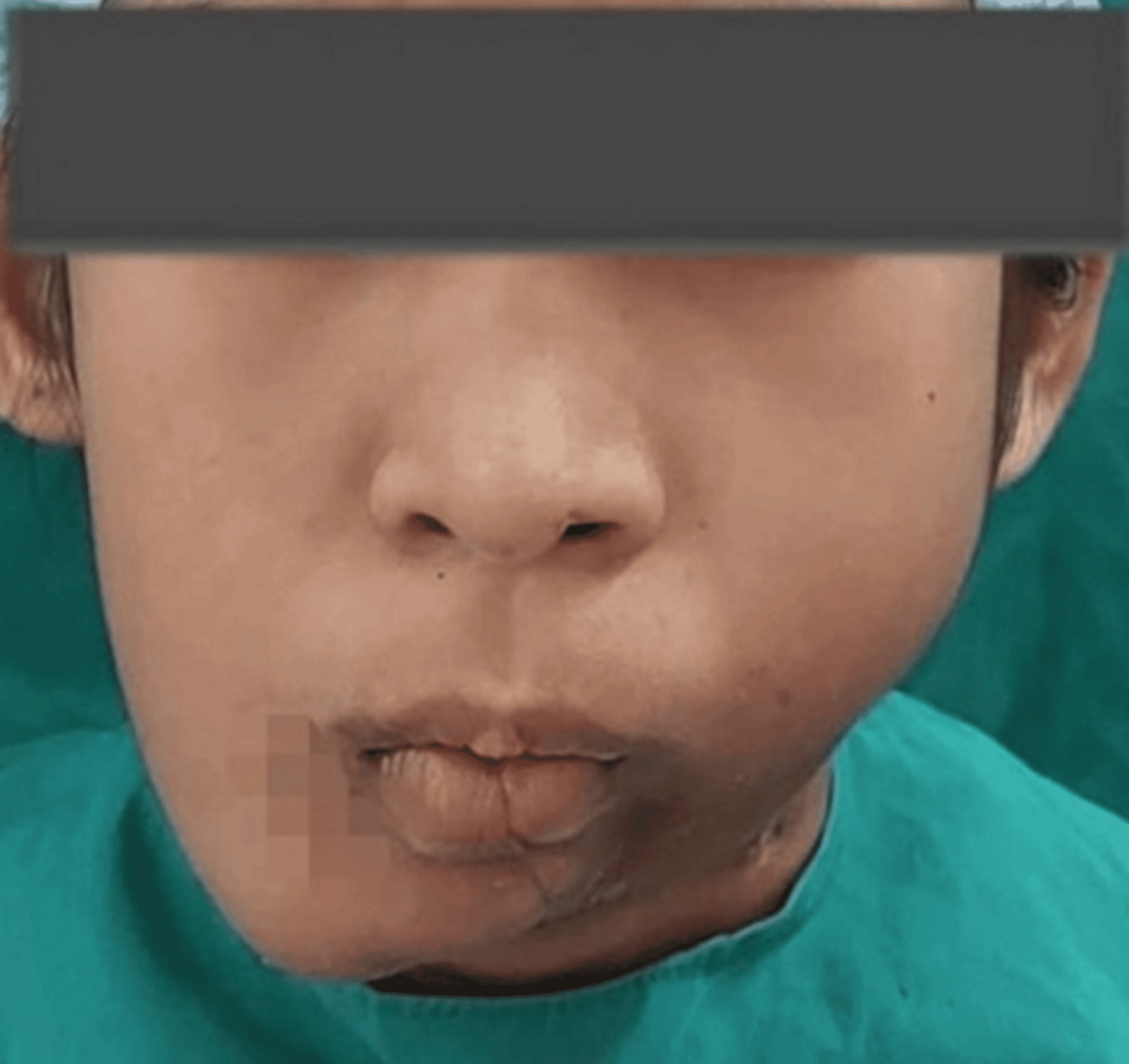
Post-operative follow-up.

Postoperative healing was uneventful, and at one-year follow-up, there was no evidence of recurrence with excellent restoration of mandibular contour (Figure 4).

Case 2

A nine-year-old male presented with a painless, slowly enlarging swelling involving the left body of the mandible over a period of eight months. Clinical examination revealed a firm, non-pulsatile swelling causing mild facial asymmetry. The overlying mucosa was intact, and the involved teeth showed mild displacement without mobility.

**Figure 5:**
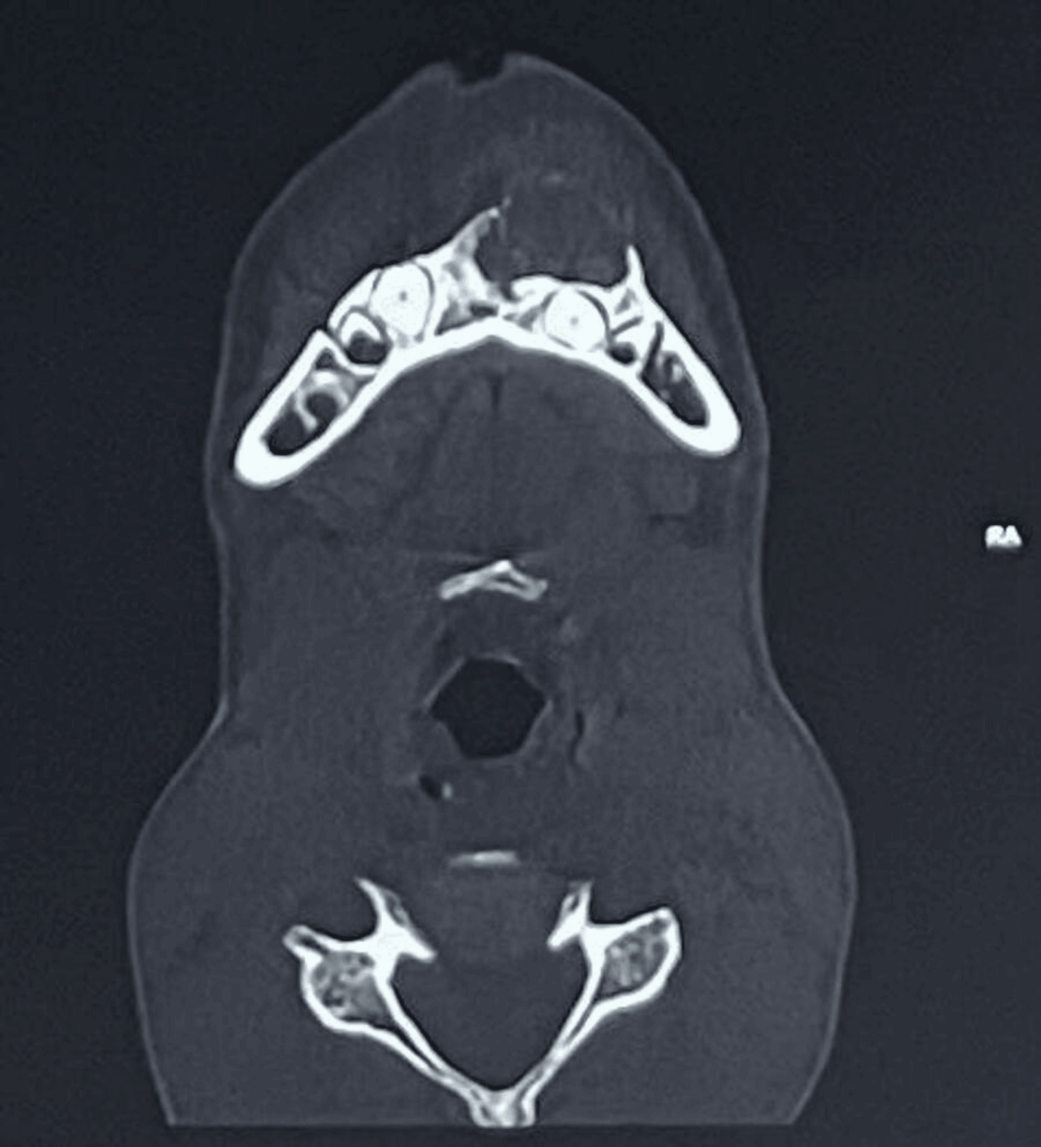
Axial view of CT scan showing an expansile intrabony lesion. Slice thickness: 1 mm at 1 mm interval.

Orthopantomogram demonstrated a multilocular radiolucent lesion with scalloped margins, mimicking ameloblastoma. Computed tomography revealed an expansile intrabony lesion with internal septations and areas of cortical perforation measuring approximately 28 × 22 × 18 mm in greatest dimensions (Figure 5).

Preoperative angiography demonstrated prominent vascular channels with feeder vessels arising from the facial artery, confirming the vascular nature of the lesion. Based on these findings, a provisional diagnosis of an intraosseous vascular lesion was made.

**Figure 6:**
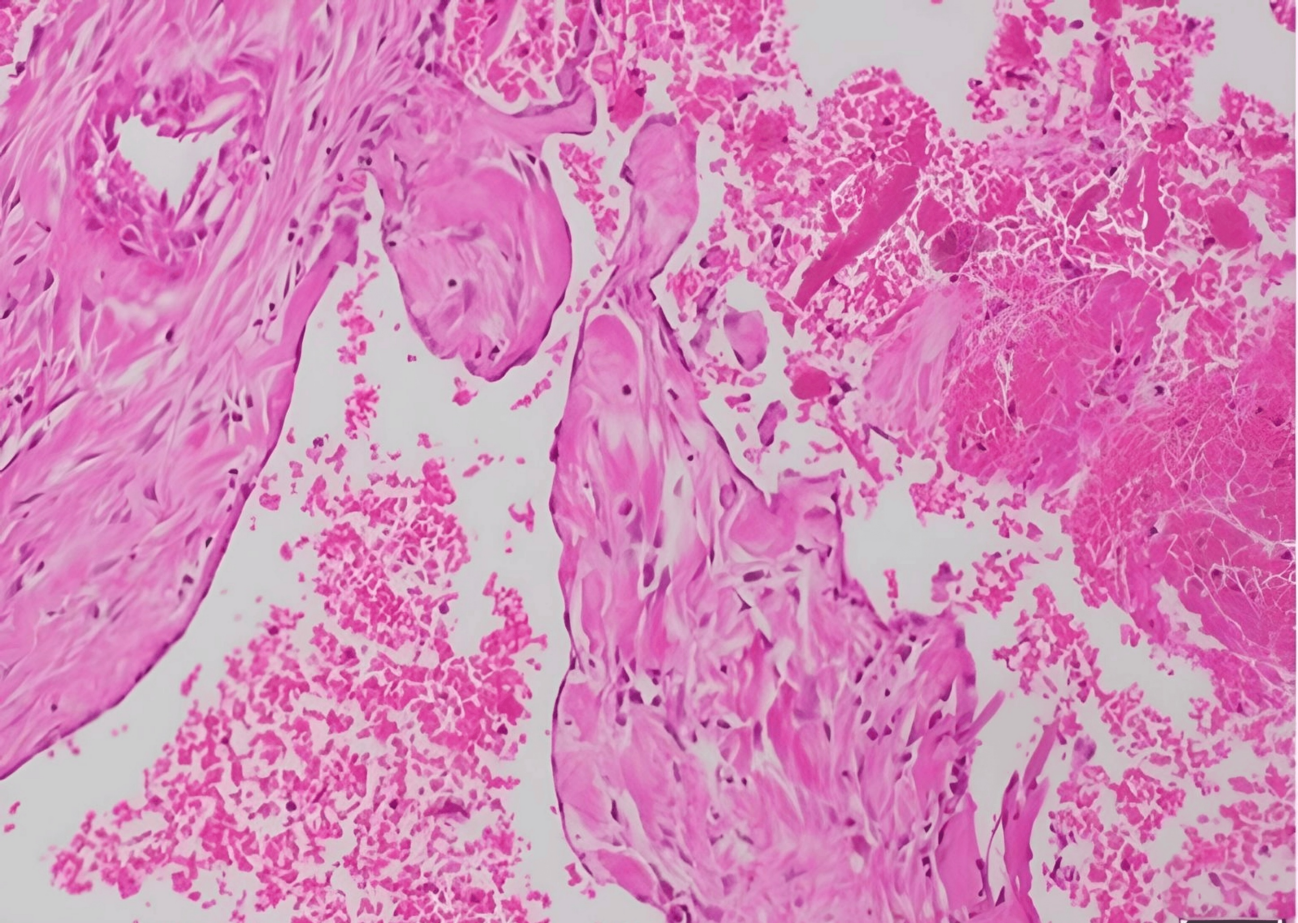
Histopathological photo demonstrating large blood-filled vascular spaces within bone, consistent with cavernous hemangioma. Magnification: 10x.

En bloc resection was performed under general anesthesia with adequate hemostatic control. Histopathological examination confirmed cavernous vascular spaces within trabecular bone, consistent with intraosseous hemangioma (Figure 6).

**Figure 7:**
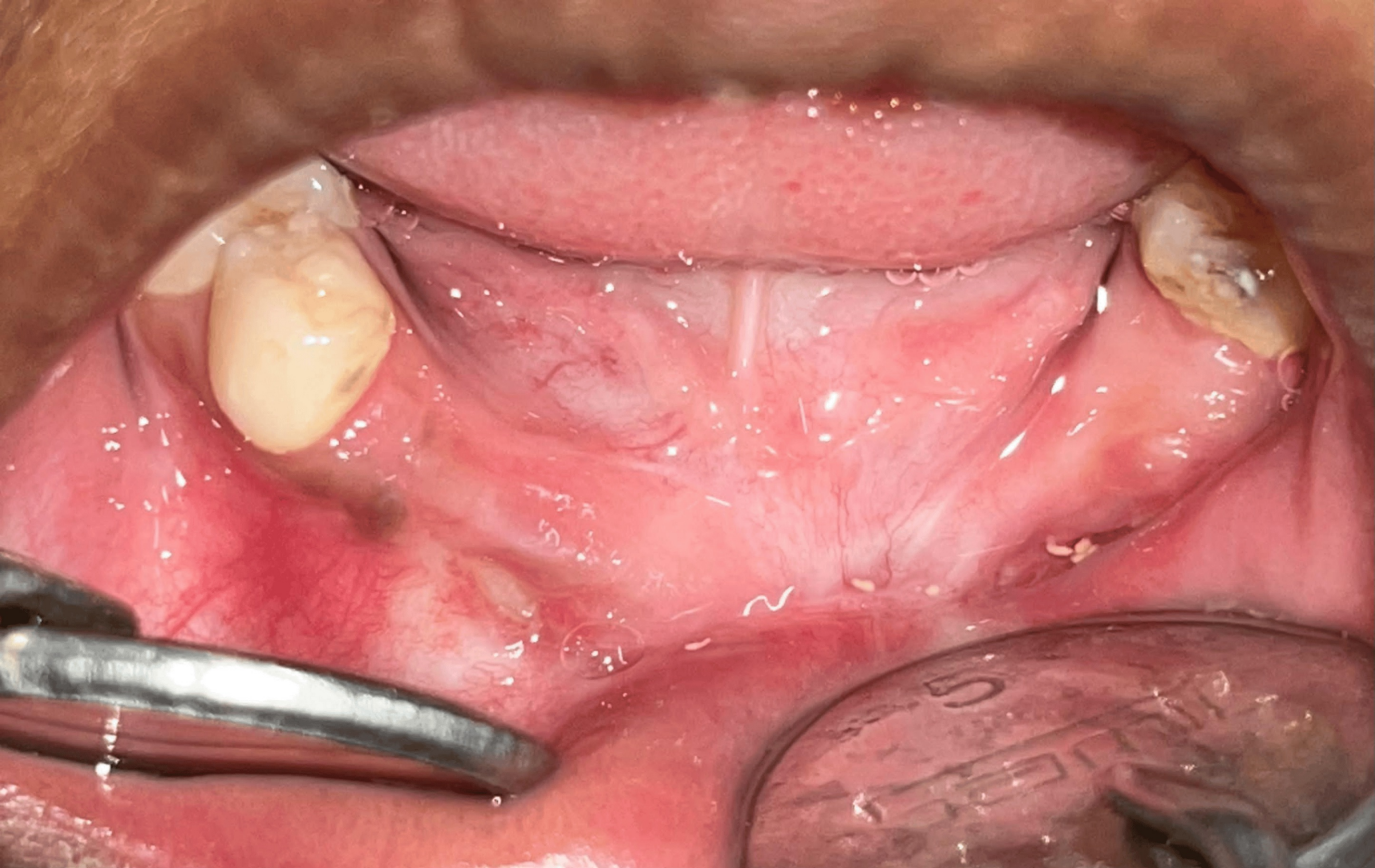
One-year post-operative follow-up.

The postoperative course was uneventful, and follow-up at one year revealed no evidence of recurrence (Figure 7).

Case 3

A 14-year-old female presented with a swelling in the left lower back tooth region of three months’ duration. The swelling was gradual in onset, progressive, and not associated with pain or discharge.

Extraoral examination revealed a well-defined swelling over the left lower third of the face, measuring approximately 50 mm × 40 mm, causing facial asymmetry. The swelling was soft to firm, non-tender, and pulsatile. Intraoral examination demonstrated diffuse swelling extending from the 34 to 37 regions with vestibular obliteration. The overlying mucosa appeared normal.

**Figure 8:**
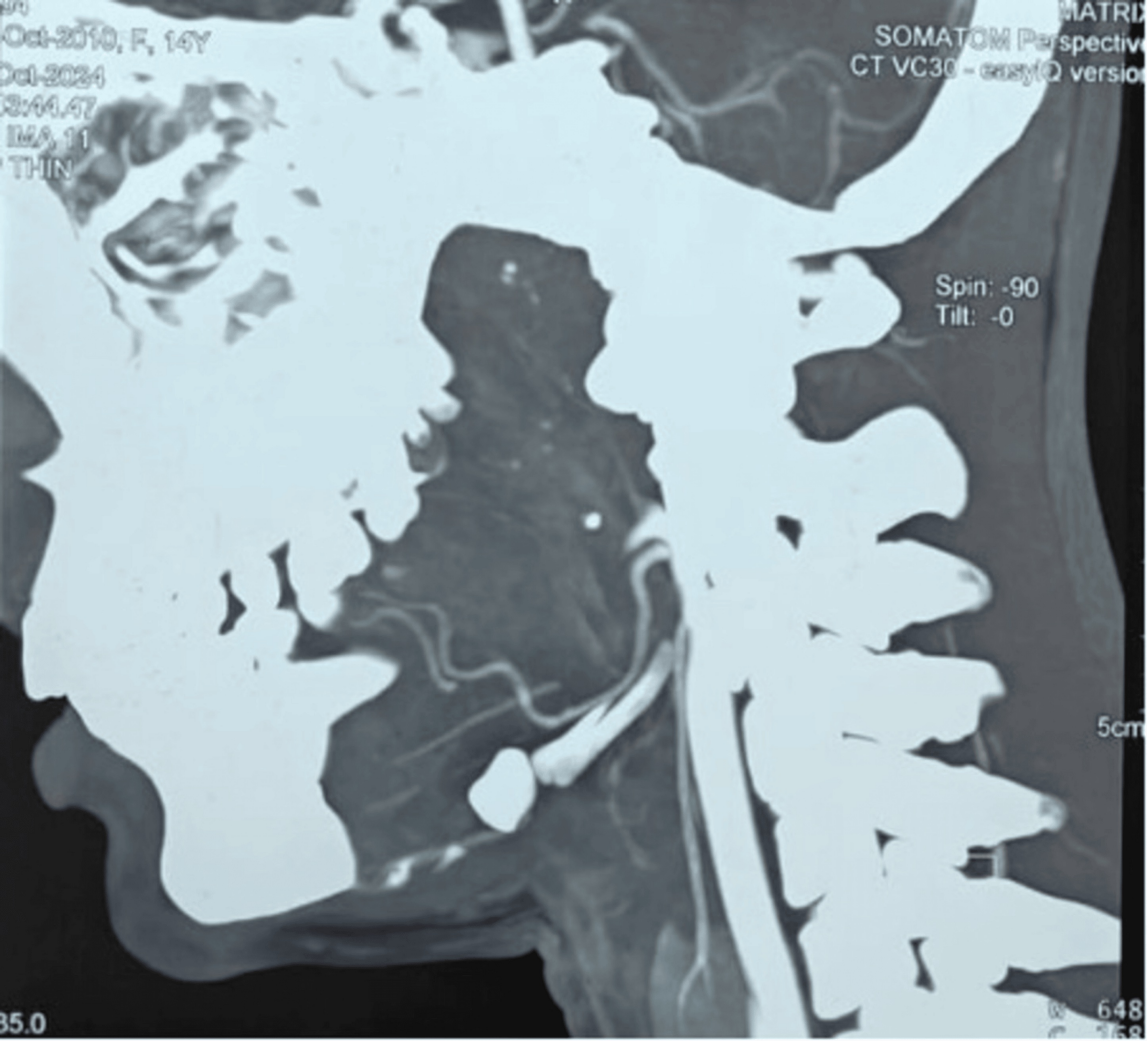
CT angiogram showing facial artery as feeder vessel. Slice thickness: 1 mm at 1 mm interval.

Aspiration yielded blood, raising suspicion of a vascular lesion. Radiographic evaluation, including orthopantomogram and computed tomography with angiography, revealed an osteolytic lesion of the mandible measuring approximately 39 × 53 × 42 mm in greatest dimensions with a hypervascular extraosseous soft tissue component (Figure 8).

**Figure 9:**
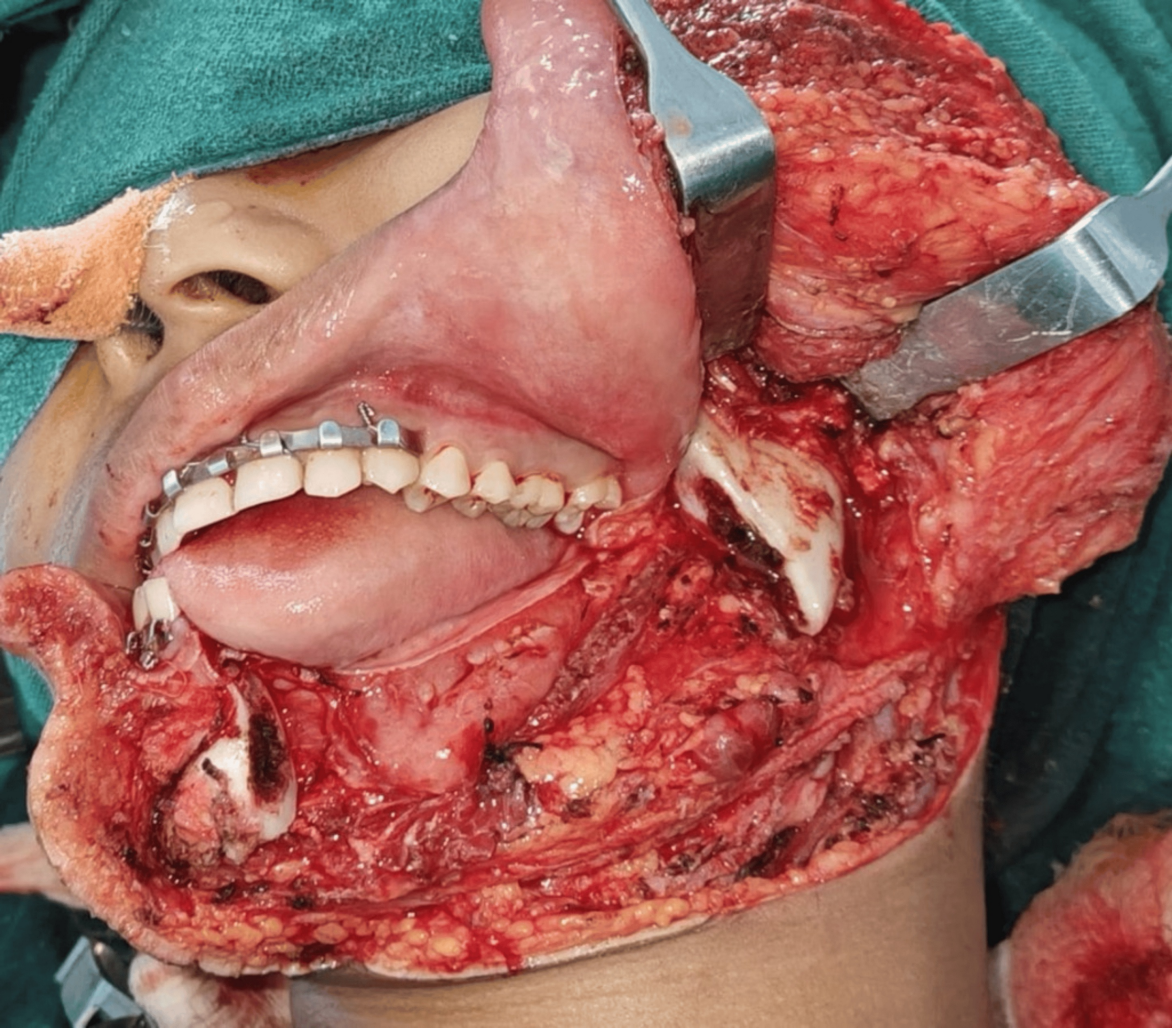
Segmental mandibulectomy defect after complete excision of the lesion.

Based on clinical and radiographic findings, a provisional diagnosis of an intraosseous vascular lesion was made. Surgical management was performed under general anesthesia. Following identification and ligation of facial vessels, segmental mandibulectomy was carried out with complete excision of the lesion (Figure 9).

**Figure 10:**
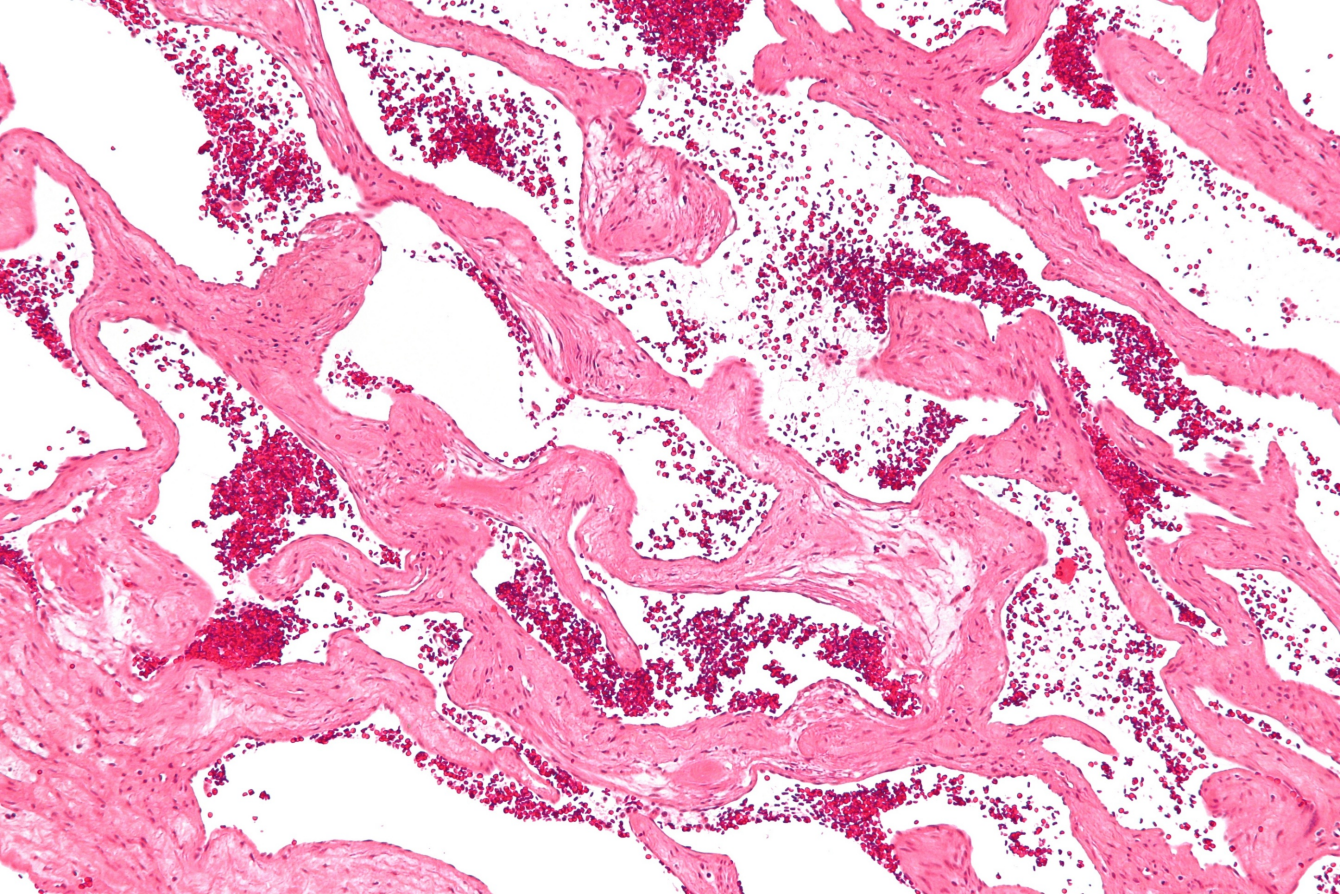
Post-operative histopathological view. Magnification: 10x.

Histopathological examination revealed large dilated vascular spaces lined by endothelial cells within bony trabeculae, confirming cavernous hemangioma (Figure 10).

**Figure 11:**
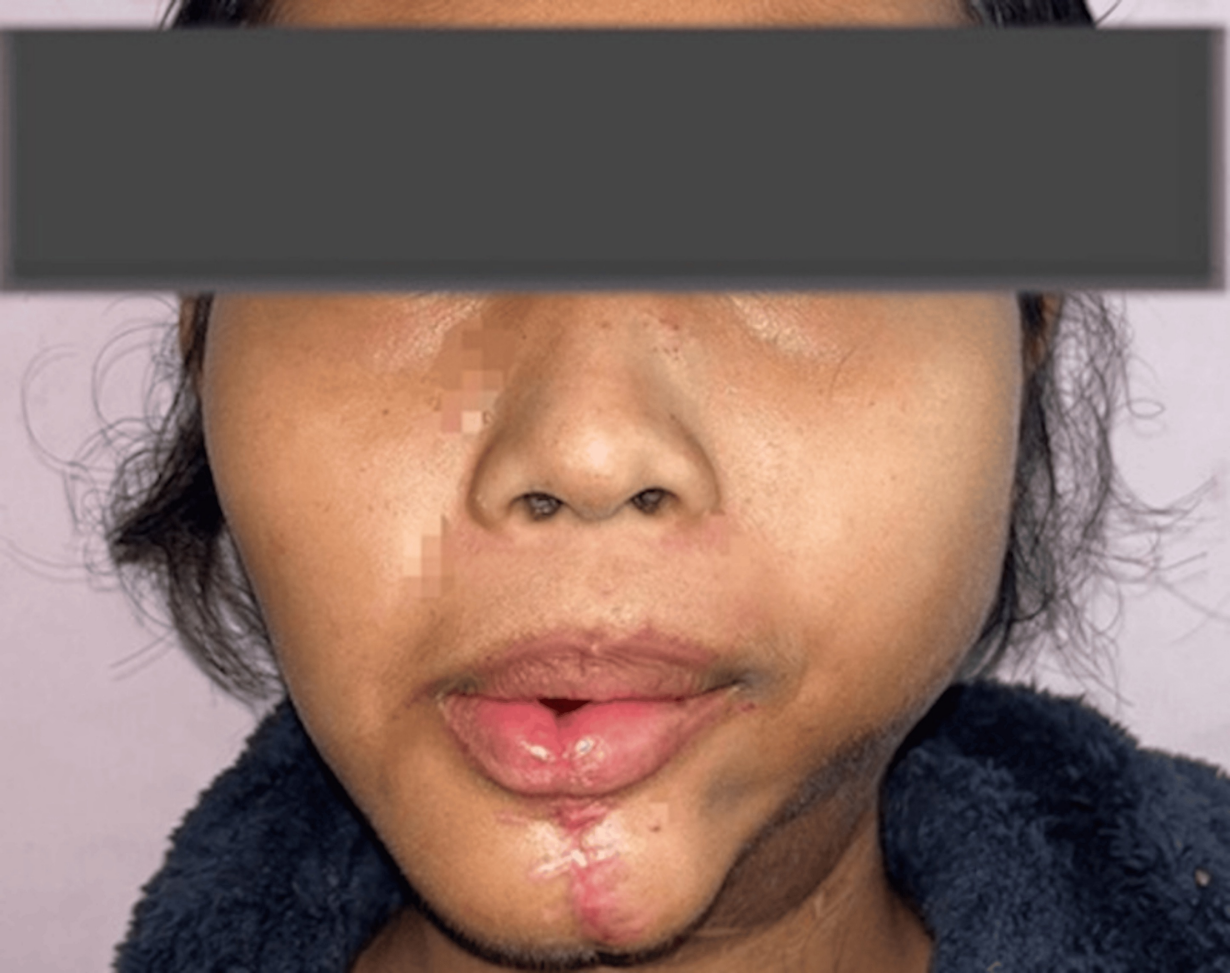
One-year post-operative follow-up.

The postoperative period was uneventful, and follow-up demonstrated satisfactory healing with no evidence of recurrence (Figure 11).

A comprehensive summary of clinical presentation, imaging characteristics, angiographic findings, operative management, and outcomes of all cases is presented in Table 1.

**Table 1:** Summary of clinical, radiological, and operative findings.

Case	Age/sex	Clinical features	Imaging findings	Angiographic feeders	Surgical approach	Blood loss	Reconstruction	Follow-up
Case 1	11/F	Pulsatile swelling, facial asymmetry	Sunburst pattern, expansile lesion	Facial artery	Segmental mandibulectomy	~1000 ml (transfusion)	Primary contour restoration	No recurrence at one year
Case 2	9/M	Painless swelling, mild asymmetry	Multilocular radiolucency	Facial artery	En bloc resection	~250 ml	None	No recurrence at one year
Case 3	14/F	Pulsatile swelling, cortical expansion	Osteolytic lesion + soft tissue component	Facial vessels	Segmental resection	~600 ml	Titanium reconstruction plate	No recurrence at one year

A summary of operative parameters, including duration of surgery, estimated blood loss, transfusion requirements, and reconstruction methods, is presented in Table 2.

**Table 2:** Operative and reconstruction details of cases.

Case	Lesion size (cm)	Duration of surgery (hours)	Estimated blood loss (ml)	Blood transfusion	Reconstruction method
Case 1	56 × 56 × 66	3	1000	Yes	Segmental resection + primary contour restoration
Case 2	28 × 22 × 18	1.5	300	No	En bloc resection
Case 3	39 × 53 × 42	2.5	700	Yes	Segmental resection + titanium reconstruction plate

## Discussion

Intraosseous hemangiomas of the mandible are uncommon lesions that often present diagnostic and therapeutic challenges due to their variable clinical and radiographic characteristics [1,4]. The present case series reinforces the heterogeneity of these lesions, which frequently leads to confusion with more common odontogenic pathologies.

Consistent with previous reports, the lesions in this series presented primarily as painless swellings with gradual progression [2,4]. However, classical vascular signs such as pulsation or bruit are not consistently observed, making clinical diagnosis alone unreliable [4]. This variability underscores the importance of maintaining a high index of suspicion when evaluating intraosseous lesions of the jaw.

Radiographic findings in intraosseous hemangioma are highly variable and may range from unilocular or multilocular radiolucencies to mixed or radiopaque patterns [4,6]. While certain characteristic patterns, such as “sunburst” appearance, have been described, they are not universally present. This variability often results in misinterpretation as lesions such as ameloblastoma or other odontogenic tumors [4]. Therefore, radiographic assessment must always be correlated with clinical findings and supplemented with further investigations.

Aspiration remains a simple yet crucial diagnostic tool in suspected vascular lesions, as it can help identify the vascular nature of the lesion and prevent catastrophic bleeding during biopsy or surgical intervention [8]. Advanced imaging techniques, including computed tomography and angiographic studies, are invaluable in determining lesion extent, identifying feeder vessels, and planning surgical management [1,9].

Management strategies for intraosseous hemangiomas depend on the size, location, and extent of the lesion. Surgical resection remains the most commonly employed treatment modality, particularly for extensive lesions, with reconstruction performed as required [5,6]. Recent advances, such as virtual surgical planning, have further improved precision and outcomes in mandibular reconstruction, allowing better functional and aesthetic rehabilitation [7]. Preoperative measures such as embolization or vascular control may be necessary to reduce intraoperative blood loss and improve surgical safety [10].

Control of intraoperative hemorrhage remains a critical consideration in the management of intraosseous vascular lesions. Various strategies have been described, including preoperative embolization and surgical ligation of feeder vessels. Preoperative embolization can effectively reduce vascularity and intraoperative blood loss; however, it requires specialized interventional radiology support and may be associated with complications such as tissue necrosis or recurrence due to collateral circulation. Alternatively, surgical ligation of feeder vessels, as performed in the present cases, provides a reliable and accessible method of hemorrhage control, particularly in resource-limited settings. Bouloux and Perciaccante [9] emphasized that both embolization and ligation are effective strategies, with the choice depending on lesion extent, vascularity, and available expertise. In our series, satisfactory hemorrhage control was achieved using ligation, with acceptable intraoperative blood loss and no major complications.

A multidisciplinary approach is essential for the effective management of these lesions. Collaboration between oral and maxillofacial surgeons, radiologists, and pathologists facilitates accurate diagnosis, appropriate imaging interpretation, and safe surgical planning. Histopathological examination ultimately confirms the diagnosis and helps differentiate hemangiomas from other vascular malformations [3,5].

The prognosis of intraosseous hemangioma is generally favorable following complete surgical excision, with low recurrence rates reported in the literature [5,10]. The present case series further emphasizes the importance of early diagnosis, careful evaluation, and a coordinated treatment approach in achieving successful outcomes.

However, this study is limited by the small sample size and the lack of long-term functional and reconstructive outcome data. Future studies with larger cohorts and standardized reporting of perioperative parameters, including operative duration, blood loss, and reconstruction outcomes, are recommended to further validate optimal management strategies.

## Conclusions

Intraosseous cavernous hemangioma of the mandible is a rare vascular lesion that can mimic common odontogenic pathologies, leading to diagnostic challenges. Early identification through aspiration and advanced imaging is essential to prevent serious hemorrhagic complications. Surgical resection with adequate vascular control provides favorable outcomes. A multidisciplinary approach involving surgeons, radiologists, and pathologists is crucial for accurate diagnosis and safe management. These cases emphasize the importance of including vascular lesions in the differential diagnosis of mandibular swellings in young patients.
